# Nutritional Guidelines and Fermented Food Frameworks

**DOI:** 10.3390/foods6080065

**Published:** 2017-08-07

**Authors:** Victoria Bell, Jorge Ferrão, Tito Fernandes

**Affiliations:** 1 Faculty of Pharmacy, Coimbra University, Pólo das Ciências da Saúde, 3000-548 Coimbra, Portugal; victoriabell1103@gmail.com; 2 The Vice-Chancellor’s Office, Universidade Pedagógica, Rua João Carlos Raposo Beirão 135, Maputo, Moçambique; ljferrao@icloud.com; 3 Associação para o Desenvolvimento das Ciências Veterinárias (ACIVET), Faculty of Veterinary Medicine, Lisbon University, 1300-477 Lisboa, Portugal

**Keywords:** fermented foods, nutritional guidelines, legislation, national food guides

## Abstract

This review examines different nutritional guidelines, some case studies, and provides insights and discrepancies, in the regulatory framework of Food Safety Management of some of the world’s economies. There are thousands of fermented foods and beverages, although the intention was not to review them but check their traditional and cultural value, and if they are still lacking to be classed as a category on different national food guides. For understanding the inconsistencies in claims of concerning fermented foods among various regulatory systems, each legal system should be considered unique. Fermented foods and beverages have long been a part of the human diet, and with further supplementation of probiotic microbes, in some cases, they offer nutritional and health attributes worthy of recommendation of regular consumption. Despite the impact of fermented foods and beverages on gastro-intestinal wellbeing and diseases, their many health benefits or recommended consumption has not been widely translated to global inclusion in world food guidelines. In general, the approach of the legal systems is broadly consistent and their structures may be presented under different formats. African traditional fermented products are briefly mentioned enhancing some recorded adverse effects. Knowing the general benefits of traditional and supplemented fermented foods, they should be a daily item on most national food guides.

## 1. Introduction

Human nutrition begins with milk. Fermented milk products have been recognized as healthy foods since ancient times. Fermentation processes and products are believed to have been developed 9000 years ago in order to preserve food for times of deficiency, improve flavor, and reduce poisonous effects. Recommendations for the consumption of certain nutritious foods date back to the Hippocratic Corpus of Ancient Greece [1]. Thousands of different fermented foods and beverages are still unknown outside the native area in which they have been produced for centuries, many going back even before recorded history [2]. Fermented foods and beverages pass through a process of lacto fermentation in which natural bacteria or yeasts feed on the sugar and starch in the food creating lactic acid.

The list of fermented products is extremely vast and the diversity derives from the heterogeneity of traditions found in the world, the cultural preference, different geographical areas where they are produced and the staple and/or by-products used for fermentation. The most popular involve beverages such as wine, beer, cider and foods such as yoghurt, cheese, soya, beans, fish, meat, cabbages, among others. In many instances, it is highly likely that the methods of production were unknown and came about by chance, and were passed down by cultural traditional values to subsequent generations [3].

Modern food is submitted to many processing methods such as pasteurization, affecting its nutritional value by reducing vitamins, fiber, minerals, essential fatty acids and amino acids. Food security can be enhanced in poor rural areas with fermented products, generating income in a small-scale family farm in developing countries [4]. The importance of fermentation is reflected by the amount and variety of foods and beverages traded not only for the benefits on nutrition and health-promoting effects but also for preservation, safety, and their peculiar appreciated sensory attributes [5].

The exploration of the microbial communities and enzymes of fermented products has been extensively reviewed [6]. At the genus level, *Lactobacillus* is usually the most abundant genus, followed by *Lactococcus*, *Enterococcus*, *Vibrio*, *Weissella*, *Pediococcus*, *Enterobacter*, *Salinivibrio*, *Acinetobacter*, *Macrococcus*, *Kluyvera* and *Clostridium*.

A better knowledge of microorganisms and fermentation at a molecular level is still required to support and develop the production of sustainable fermented food with high nutritional characteristics. A metagenomic approach has enabled identification of novel microbiome profiles and exploration of microbial compositions in a range of traditional fermented foods while bypassing the need for cultivation, allowing the identification of a vast array of microorganisms never previously isolated in culture [7].

## 2. Sustainable Development and Fermented Food Hygiene in Africa

Food preservation increases the range of raw materials and by-products that can be used to produce edible food products and remove anti-nutritional factors, rendering food safe to eat by humans and animals. Fermentation is a cheap way of preserving perishable raw materials, accessible to even the most marginalized people. Utilizing small-scale fermentation contributes to economic and social benefits and sustainable development of families and communities [8]. Poor hygiene or improper post-handling fermentation limits shelf-life and becomes dependent on information from developed countries and technology transfer [9].

Regulators will only be convinced on causal relationships existing between fermented foods/beverages and health benefit or eventual risk, through the development of scientific dossiers, which is only feasible by industrialized producers [10].

Fermented foods may be recommended for improving the health and nutritional quality of traditional African foods and regular inclusion of fermented products as part of the daily diet would be desirable. However, lack of knowledge and understanding toward fermented food preparation may limit their usage.

Hazard Analysis and Critical Control Point (HACCP) studies in Africa of some fermented products have demonstrated that, depending on the process and the hygienic conditions observed during preparation, some fermented foods, e.g., *togwa*—fermented cereals prepared in Tanzania—may pose a safety risk [11].

Accidents may occur. For example, in 2015, at least 75 people died and some 180 fell ill, including a toddler, in the north-west of Mozambique, from apparent poisoning after consuming during a wedding party traditional fermented beer (made of sorghum, bran, corn, sugar, with *Schizosaccharomyces pombe* yeast, which belongs to the division Ascomycota, which represents the largest and most diverse group of fungi) known as “*pombe*” (Swahili word for beer). The exact cause of the contamination was later connected with bacterium *Burkholderia gladioli* and two produced toxins, bongkrekic acid and toxoflavin [12].

These safety concerns relating to pathogenic bacteria or chemical intoxicants produced by contaminating microorganisms, yeasts or moulds on a fermented food were also demonstrated by the deaths and risks of esophageal cancer reported by the consumption of fermented milk products from Kenya including *Mursik* (a cow or goat´s milk fermented in a calabash gourd), *Kule naoto*, *Amabere amaruranu* and *Suusa* [13,14,15].

## 3. Fermented Foods and Probiotics

Fermented foods belong to a category of foods called “functional foods that are known to have a positive effect on health” [16]. Probiotics are the bacteria used to ferment traditional foods, and they are the most reported and researched. Thus, fermented foods and probiotics are closely related and co-exist despite the increased commercial interest in probiotics due to the health attributes associated with them [17]. However, the efficacy of probiotics is enhanced when taken in the form of fermented food rather than as probiotics alone [18].

Microbial food cultures have directly or indirectly come under various regulatory frameworks in the course of the last decades. Several of those regulatory frameworks put emphasis on “the history of use”, “traditional food”, or “general recognition of safety”. Traditionally fermented foods are highly beneficial because they supply natural probiotics, now recognized as crucially important for immune health. Fermentation is an inconsistent process—almost more of an art than a science—so commercial food processors developed techniques to help standardize more consistent yields [19].

Fermentation is an anaerobic process converting sugars by bacterial enzymes to alcohol or by yeasts into lactic acid. Fermented foods are described as palatable and wholesome and are generally appreciated for several attributes: their specific unique flavors, aromas, textures, and improved cooking and processing properties. These characteristics of fermented foods are enhanced by virtue of the metabolic activities of the enzymes secreted by microorganisms [20].

Probiotics incorporate mainly fermented dairy foods. While EU permits animal production claims for feed probiotics, the USA and Canada do not. Regulators now accept several modes of action of probiotics, not just gut flora modulation [21], increasingly demanding safe strains [22]. An inventory of microorganisms used in food fermentations covering a wide range of food matrices is also required (dairy, meat, fish, vegetables, legumes, cereals, beverages, and vinegar) [23].

Various indigenous fermented foods containing probiotic bacteria have been part of local diets in Africa due to reported medicinal properties they possess [24]. Consumers must be made aware of the problems concerning raw materials and additives used in food and beverage processing as well as the possible harmful effects of employing genetically modified microorganisms in the fermentation process.

Further development of traditional fermented foods with added probiotic health features would be an important contribution towards reaching the goals of eradication of poverty and hunger, reduction in child mortality rates and improvement of maternal health in Africa [25]. Probiotic consumption may have a positive effect on psychological symptoms of depression, anxiety, and perceived stress in healthy Western human populations [26].

Fermented foods have been inching into the spotlight lately as more and more consumers learn about their inherent probiotic health benefits. The two main health effects from fermented dairy consumption are immune and metabolic positive responses, especially with the addition of probiotic organisms.

## 4. Public Policy: Regulations, Laws, Opinions and Guidelines

The General Food Law Regulation in the European Union (EC No. 178/2002) establishes the European Food Safety Authority (EFSA) and lays down procedures in matters of food safety, including later the principle of risk analysis.

Access to sufficient and safe food is a basic requirement for human health. In the past decades, the increased complexity of the food supply chain has contributed to the global emergence of food safety incidents. Indeed, over the past decades, a series of food safety incidents (e.g., BSE- Bovine Spongiform Encephalopathy in the 90’s) have shown serious shortcomings in legal systems worldwide (e.g., law enforcement, lack of appropriate response mechanisms, control systems) [27].

**Figure 1:**
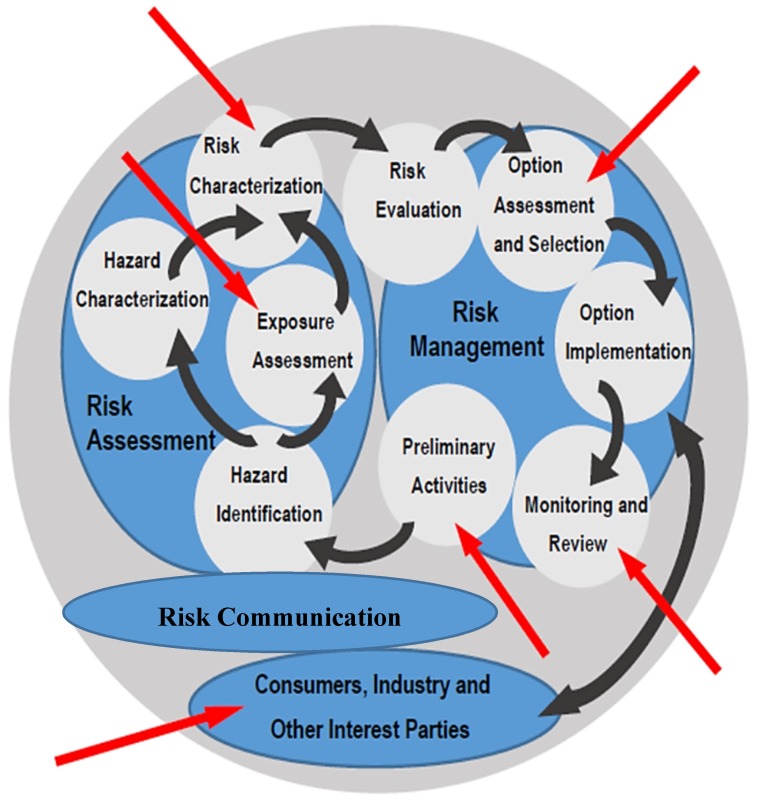
Integration of risk evaluation, communication and management by the EFSA.

It was the lack of risk communication and risk management (comprehensive/integrated approach) that brought the weaknesses of the legal food system to light and provided a new approach to food safety emerged (Figure 1) [28,29].

In addition to legislation, producers of traditional fermented food and beverages are also being increasingly obliged to meet various legislative requirements. These may take various forms including taxes and “certification of origin”.

The challenges of implementing measures to safeguard safety and quality in the real world are numerous. There is a notable role for ongoing training and education of all those involved as to the importance of quality and standards not only for successful trade, but also in terms of social responsibility [30].

The U.S. Food and Drug Administration (FDA) published (September 2010) the 75FR50268, “*Draft Guidance for Industry: Acidified Foods*”, which provided recommendations on manufacturing, storage, packaging, distribution processes, and appropriate quality control procedures for acid foods, acidified foods, and fermented foods. However, in December 2015 (80FR81550), they withdrew partially the draft guidance because many of the topics addressed are presently being dealt with in other documents. The FDA does not regulate fermented foods since they have not found any cases of food illnesses [31]. A comparison of key elements between EU and the USA is given below (Table 1).

## 5. The Case of Fermented Soybean Extracts in Europe

Japanese cuisine, one of the healthiest foods in the world, reflected in the high life expectancy (83 years), is known to use fermented foods such as “Natto” which is sticky, smelly and slimy but still the most popular of Japan’s traditional health foods.

Japan Bio Science Laboratory (JBSL) submitted in Europe a request on May 2014, under Article 4 of the Novel Food Regulation (EC No. 258/97) [32], to place on the market a fermented soybean extract (Nattokinase NSK-SD^®^), obtained by adding beneficial bacteria *Bacillus natto* to soybeans, as a novel food (NF). On December 2014, the competent authority of Belgium forwarded to the European Commission its initial assessment report, which came to the conclusion that the fermented soybean extract met the criteria for acceptance of a NF defined in Article 3 (1) of Regulation (EC) No. 258/79. On 6 January 2015, the Commission forwarded the initial assessment report to the other Member States (MS).

However, several of the MS submitted comments or raised objections. The concerns of a scientific nature raised by the MS on this fermented food can be summarized on their effects on hematological parameters, in particular on blood coagulation. One case report [33] was identified, in which a patient on antihypertensive agents and low-dose aspirin for secondary stroke prevention experienced an acute cerebellar hemorrhage and cerebral micro bleeds after concomitant consumption of this fermented soybean extract.

In accordance with Article 29 (1) (a) of Regulation (EC) No. 178/20024, the European Food Safety Authority (EFSA) was asked to carry out an additional assessment for the fermented soybean extract as an NF in the context of Regulation (EC) No. 258/97. The EFSA considered the elements of a scientific nature in the comments raised by MS and a Scientific Opinion was produced [34] concluding that the fermented soybean extract was safe under the intended conditions of use as specified by the applicant.

## 6. The Case of Gluten and Celiac Disease

Another good example is Celiac disease, a hereditary, chronic inflammatory disorder of the small intestine, which has no cure, associated with gluten intake. In the U.S. Federal Register [35], a final rule was published defining the term “gluten-free” and establishing the requirements for the voluntary use of that term in food labeling. The final rule (21 CFR 101.91) is intended to ensure that individuals with celiac disease are not misled and are provided with truthful and accurate information with respect to foods so labeled.

There is still uncertainty in interpreting the results of test methods on a quantitative basis that equates the test results to an equivalent amount of intact gluten. Therefore, alternative means are necessary to verify compliance with the provisions of the rule for fermented and hydrolyzed foods, such as cheese, yogurt, vinegar, sauerkraut, pickles, green olives, beers, and wine, or hydrolyzed plant proteins used to improve flavour or texture in processed foods such as soups, sauces, and seasonings.

## 7. The Homemade Foods Bill in the USA

A regulation (HB 1926) in Texas addresses genuine concerns about the risks of the food and expanded distribution. The bill addresses food safety concerns in a scale-sensitive manner, allowing for safe home food production and sales. This benefits not only producers, but also consumers, who receive improved access to healthy, locally produced foods while still providing realistic opportunity for home production, allowing home preparation of foods such as tamales, canned vegetables, fermented foods, and perishable (potentially hazardous) baked goods. Sales would be allowed anywhere in the state, including through mail order and internet sales, as long as the producer and consumer were both in Texas.

Farmers who have unsold vegetables at the end of a farmers’ market may send those vegetables to their customers, and home bakers can work with grocery stores or other retail outlets to sell their goods giving a huge boost for small businesses. Home processors would be subject to regulatory provisions.

A new Homemade Food Law in California (AB 626), the largest agricultural producer and exporter in the United States, was introduced in 2017 but may ultimately be designed to meet the needs of big tech companies above the needs of home consumers and other stakeholders [36].

## 8. Fermented Foods and Mycotoxins

Food contamination with mycotoxins is a major problem in Africa. There is no doubt that fermentation and its products have lots of benefits. However, the fact still remains that some of the microorganisms used in the fermentation of food may become harmful under certain undesirable conditions. Microbial spores and mycotoxins such as those associated with *Aspergillus flavus*, *Aspergillus oryzae*, *Penicillium roqueforti* and other fungal toxins, such as those associated with *Fusarium*, are known to be lethal at moderate to high dosage. These toxins are produced when fermentation conditions are compromised and poor hygiene of food sources for fermentation persists during production, as happens in many African countries. *Clostridium botulinum* is an example of bacteria that causes poisoning in fermented foods and could be quite hazardous. Post fermentation contamination of products may affect the physiology of the products thereby becoming disruptive to health and deleterious to life. In order to prevent mycotoxin contamination of a fermented food, it is necessary to use a mycotoxin-free raw material and to prepare it in good sanitary and hygiene conditions [37].

## 9. Nutritional Guidelines and Consumer Expectations

Nutritional guidelines around the world come in many different formats, illustrated as pyramids, pie charts, text and tables, yet they are similar in terms of content [38]. Despite the wide spectrum of shapes representing food guides from around the globe, these guides use very similar methods in presenting their concepts of the ideal dietary pattern [39]. Each of these guidelines gives consumers a selection of recommended food choices (food groups) as well as a recommended daily amounts that consumers should ingest to maintain optimum health [40].

The present short review tries to provide some insights into the existing regulatory framework of Food Safety Management of some of the world’s economies. Although these systems do not only differ significantly with regard to their legal culture (traditional) and legal historical background, there is a strong variation of their socio-cultural background and traditions. For now, one must exclude fermented fish from Food Guides since in parts of China an increased risk of squamous cell carcinoma of the esophagus was recorded in habitual consumers of fermented fish sauce [41] or high levels of histamine, as demonstrated in Egypt [42].

Contrary to European and USA legal systems, which are relatively young, China’s legal system knows a long history that can be traced back to 563 BC based on morality. On April, 2015, the Standing Committee of China’s National People’s Congress revised the 2009 Food Safety Law of the People’s Republic of China (Food Safety Law). The revised law came into effect on October 2015.

European Law itself is not a national legal system as Chinese Law is. Neither does it relate to a common federal law system such as in the USA. In Europe, each country has its own national legal system. In practice, discrepancies in food safety incident response can be identified in the way these legal systems deal with food safety incidents, and, as a result, in the development, design and characteristics of the regulatory framework at stake.

In general, the approach of the legal systems is broadly consistent with the assessed International (FAO/WHO) framework channeled through the Codex Alimentarius Commission (CAC) in their work to develop international food standards and guidelines [43].

Europe’s food safety policy is characterized by its legal embedded preventive approach [44]. Moreover, another interesting aspect that should be taken into account in the matter of legal culture in relation to Europe is the concept of “cultural pluralism” and the characterizations thereof in the field of Food Safety Law. The “International Commission for Research into European Food History” was founded in Germany in 1989, and it deals with the history of food and nutrition in Europe since the late eighteenth century [45].

In Russia, there are “Requirements for Ferments and Enzyme Preparations” (article 12) and “Requirements as to Facilities for Ferment and Probiotic Microorganism Production” (articles 13 and 26) within a very comprehensive Federal Law [46], where the need of Fermented Food for Mandatory Certification of ready to use foods or Conformity Declaration of raw materials is clear.

## 10. Some National Food Guides

**Figure 2:**
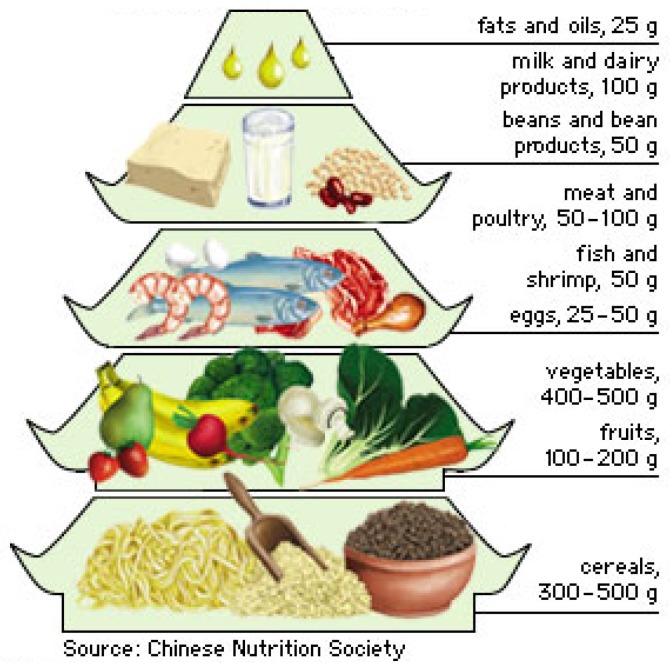
Food guide pagoda.

The Chinese dietary guidelines were first published in 1989 and revised in 2007. China uses the ‘Food Guide Pagoda’ (Figure 2), which is divided into five levels of recommendations.

**Figure 3:**
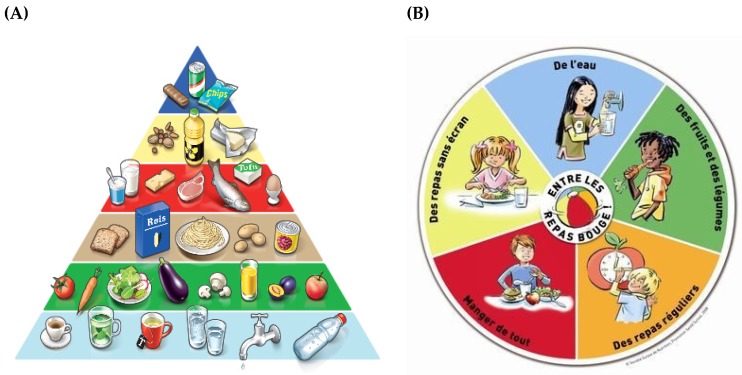
The Swiss food pyramid and children’s nutrition disk (**A**), the pyramid; (**B**), the plate for children.

In Switzerland, in order to reduce trade barriers with the European Union, the Swiss food law has been adapted to the European law (Figure 3). Some Swiss special provisions for the reason of health protection persist. For example, a positive list of health related allegations for food and the obligation for self-supervision, and reporting and licensing requirements [47].

**Figure 4:**
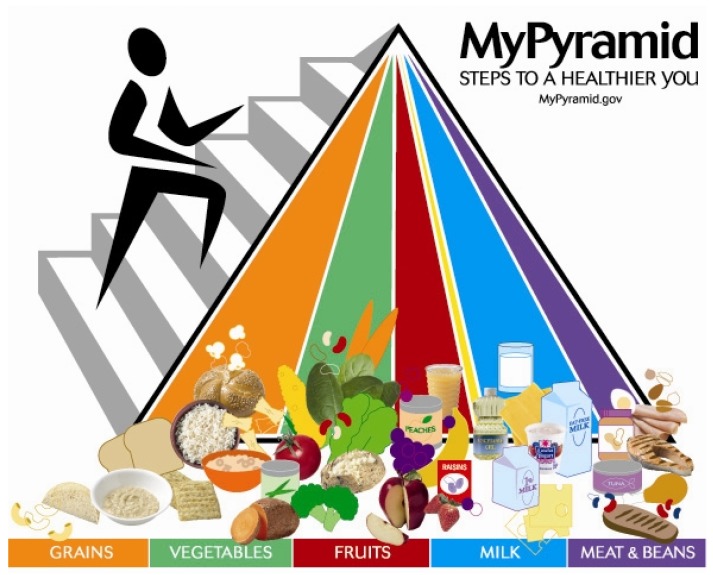
The USA MyPyramid food guide. USDA, Unites States Department of Agriculture.

**Figure 5:**
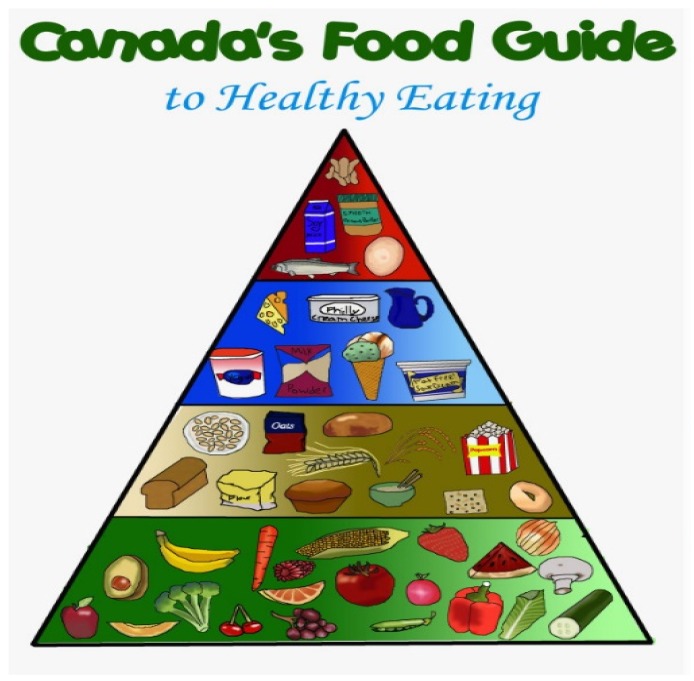
The Canadian food guide.

In the USA (Figure 4) and Canada (Figure 5), food guidelines mention yogurt and kefir, but there is no emphasis on them being fermented foods, nor is there inclusion of fermented foods as a healthy category [48].

**Figure 6:**
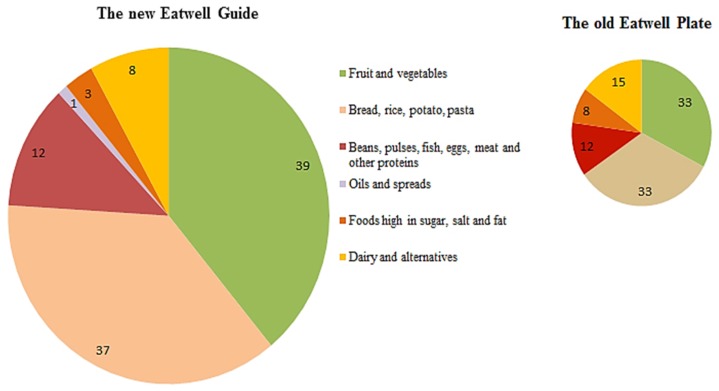
The UK food guide. Comparison with the previous food guide.

The United Kingdom (Figure 6) published its first set of dietary guidelines in 1994, and they have been regularly updated since then. Their Food Guide is still presented as a plate, and there is no category of fermented food. The “Eatwell Guide” (2017) has been recently approved [49].

**Figure 7:**
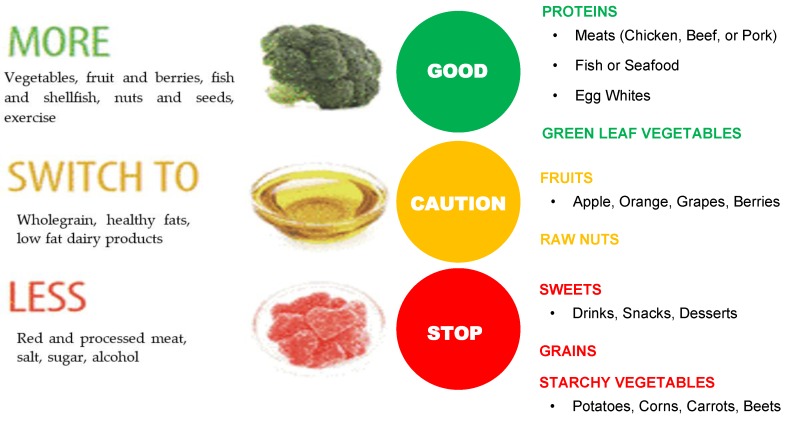
The Scandinavian food guide.

The Swedish and Norwegian models (Figure 7) for healthy eating, also in the form of a plate, have no section allotted to dairy products or any fermented foods [50,51].

**Figure 8:**
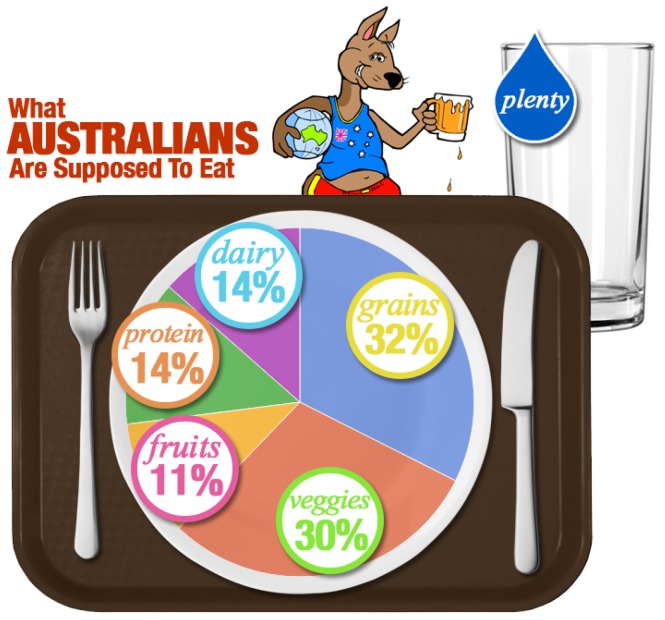
The Australian food guide.

The Australia New Zealand Food Authority produced Standards Code in 2014 (Figure 8), the standard related to Fermented Milk Products, including yoghurt; however, this was repealed in March 2016 (Table 2). Phytosterol, phytostanols and their esters may only be added to yoghurt in defined clear conditions of package size (200 g), percentage fat (1.5%) and total sterol added (the total plant sterol equivalents content added should be no less than 0.8 g and no more than 1.0 g per package) [52].

**Figure 9:**
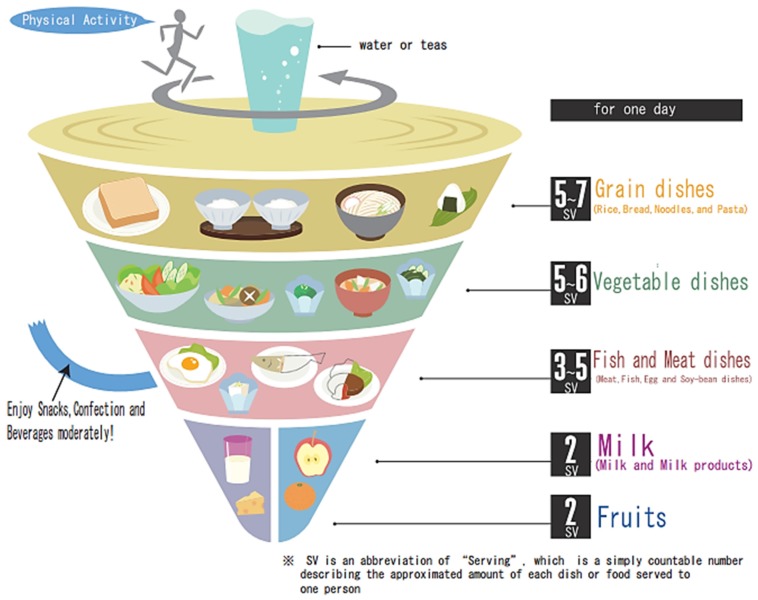
The Japanese food guide. SV= servings.

In Japan, like in most countries, it is enhanced that every food group should be taken daily in moderation in order to achieve a well-balanced diet, but it does not specifically highlight fermented foods as a category (Figure 9) [53].

**Figure 10:**
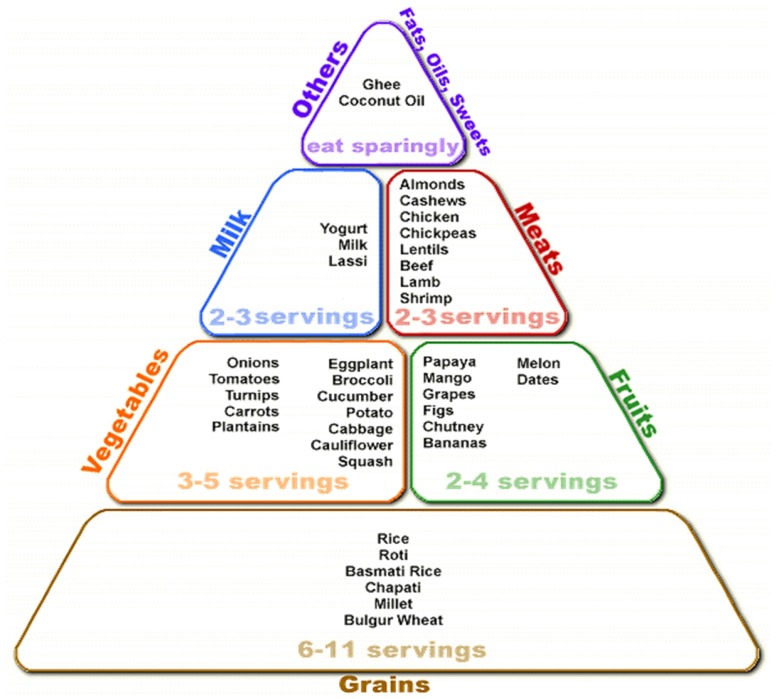
The Indian food guide.

The one exception in Asia is India, whose pyramid of four levels explicitly encourages the consumption of fermented foods (Figure 10). The National Institute of Nutrition’s 2010 “Dietary Guidelines for Indians” suggests specifically to pregnant women that they should eat more whole grains, sprouted grams and fermented foods [54].

With few exceptions, fermented foods are generally absent as a recommended category of food for daily intake in Food Guides, reflecting a failure to appreciate the benefits resulting from the process of fermentation, which have been supported by numerous studies [55].

Findings in The Netherlands, Sweden and Denmark emphasize the need to differentiate the types of dairy products, fermented and non-fermented, with regards to their health benefits, instead of promoting all dairy products, as is the case with many food guides [56,57,58].

The primary expectation of the general consumer today is that governments make sure proper measures are in place to ensure food sold is safe to eat. Hence, food is something we all consume; therefore, the safety of food is an issue valued and to which attention is widely drawn. To this end, several factors influence how an event is approached including the number of people ill, the severity of the illness, the distribution and volumes of food, whether the contaminant is known or unknown, and the international and trade implications.

Moreover, how people react to risks is mediated by many factors, including how risk information is perceived, how we react to social and cultural influences and how choices are structured [59], and the latter influences the legal construction designed to manage risks. What might be handled as a routine incident in one country may be considered a crisis in another [60].

**Figure 11:**
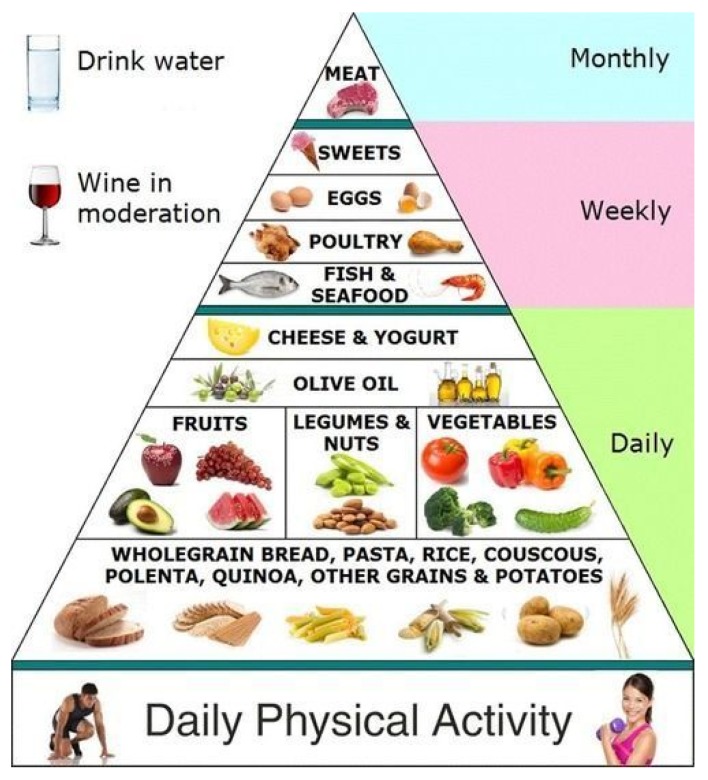
The Mediterranean diet pyramid.

There are many alternatives for reinforcing healthy eating, such as the Mediterranean, Latin and Asian Pyramids. They use specific cultural eating patterns to offer evidence-based advice for healthy eating. A traditional Mediterranean diet (Figure 11), considered as the best balanced diet to follow, is high in fruits, vegetables, nuts, unrefined grains and olive oil, with a moderate intake of fish, alcohol, and a low intake of meat and dairy products, which is inversely associated with total and cardiovascular mortality [61].

**Figure 12:**
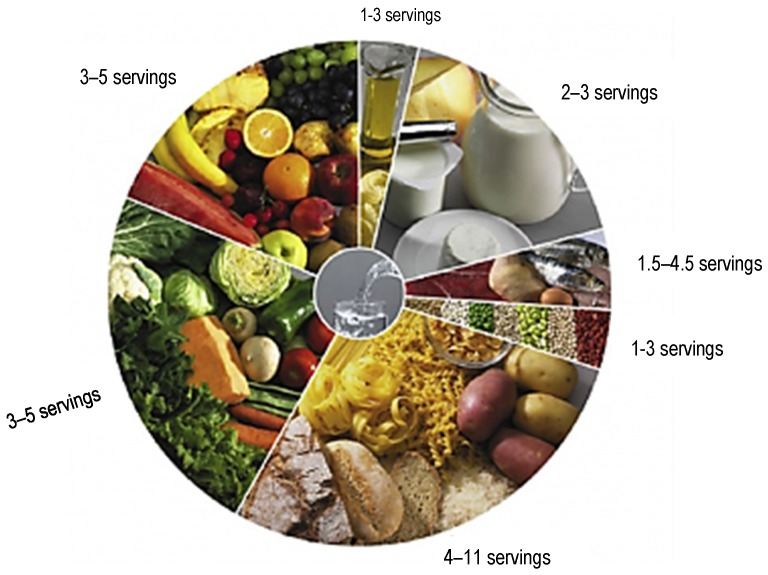
The Portuguese food wheel guide.

The Portuguese Food Guide, issued in 1977, revised in 2003, is a food wheel divided into segments representing seven food groups (Figure 12). Water is in the center of the Food wheel in order to highlight the importance of hydration balance [62].

## 11. A Comparison of Inclusion of Fermented Products in Different Guides

Evaluating dietary guidelines from various countries can help identify their strengths and limitations, yet such assessments are lacking due to the complexity involved. Trying to compare qualitatively current population-level dietary recommendations and pictorial food guides issued by government or nutrition agencies across nutrition transition stages (early, ongoing, and transitioned) is almost an impossible task.

The recommendations for consumption of foods or dietary practices within main categories of food groups, nutrients, or beverages, including lifestyle, nutritional, cooking, or eating habits is an intricate process. Some 55 major nutrients are reasonably well characterized and their required levels of intake calculated; however, the subject becomes quite complex if taking into account the amount of phytonutrients involved in plants (over 100,000)—one of the reasons nine servings of fruits and vegetables a day are recommended. Milk or fermented milk products may block the absorption of phytonutrients, some of which are considered essential.

Below (Table 3) we attempt to provide a short comparison summary among few countries.

## 12. Conclusions and Recommendations

The present global regulatory framework is confusing and limiting. Science is developing and many exciting new technologies will continue to transform the world and improve human welfare. The market is imaginative and not discouraged by the limitations of the science.

Foods prepared by fermentation, aside from those well known in the West, will increase in amount and use spreading to other parts of the world, including the developed Western countries, as they contribute to the diversity of gut microbiota and indirect impact on mental health. The knowledge about the microbial ecology of food and beverage ecosystems is essential in order to understand the production process.

Over the past decades, the increased complexity of the food supply chain has contributed to global emergence of food safety incidents. As a result, reform of Food Safety Management regulation throughout the food supply chain has been gaining momentum in legal systems worldwide. To this end, discrepancies in food safety incident response can be identified amongst legal systems. The reasons for these discrepancies reside in different models for prediction of permissible levels, non-complete information about risks and in differences in sampling procedures. The very notion of risks, as well as the understanding and evaluation of particular risks, reflect and shape the values, preferences and prejudices of a society and therefore country differences.

The FDA in the USA and the EFSA in Europe acknowledge that many proposed rules, if finalized, may have a significant economic impact on a substantial number of small entities and may impair fair and open trade among different continents and countries. Many challenges still remain regarding the establishment of dietary guidelines integrating education, agriculture, health, environment and industry.

## Figures and Tables

**Table 1:** Comparison key elements of the EU–US food regulatory systems.

In Brief	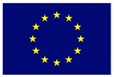	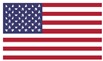
“Precautionary principle”	Fundamental part of risk management	Concept not endorsed as a basis for policy making
Societal, economic, ethical or environmental concepts	Taken into account in risk management decision in line with consumer right to information and choice	”Other factors” considered as barriers to trade
Approach to ensuring food safety	Integrated “farm-to-fork” approach	Safety mostly verified at the end of the process
Food risk evaluation	Full scientific assessment by the EFSA for regulated products such as GMO’s and additives	Largely relies on companies’ own private assessment

EFSA, European Food Safety Agency; GMO, Genetic Modified Organisms.

**Table 2:** In fermented milk and the fermented milk portion of a food containing fermented milk, each component or parameter must comply with the value specified.

Component or Parameter	Value
Crude protein	minimum 30 g/kg
pH	maximum 4.5
Microorganisms used in the fermentation	minimum 10^6^ cfu/g

**Table 3:** Fermented products within food-based dietary guidelines in some countries.

Country	Yoghurt (Included in Dairy Products)	Alcoholic Fermented Beverages
China	-	-
Switzerland	+	in moderation
USA	+	in moderation
Canada	+	-
UK	+	-
Australia	+	in moderation
Japan	+	-
Sweden	+	-
Portugal	+	in moderation

+, existent; - non existent.
